# Treadmill Training in Multiple Sclerosis: Can Body Weight Support or Robot Assistance Provide Added Value? A Systematic Review

**DOI:** 10.1155/2012/240274

**Published:** 2012-05-30

**Authors:** Eva Swinnen, David Beckwée, Droesja Pinte, Romain Meeusen, Jean-Pierre Baeyens, Eric Kerckhofs

**Affiliations:** ^1^Vakgroep KINE, Faculty of Physical Education and Physiotherapy, Vrije Universiteit Brussel, Laarbeeklaan 103, 1090 Brussels, Belgium; ^2^Research Group Advanced Rehabilitation Technology and Science (ARTS), Vrije Universiteit Brussel, Pleinlaan 2, 1050 Brussels, Belgium

## Abstract

*Purpose*. This systematic review critically analyzes the literature on the effectiveness of treadmill training (TT), body-weight-supported TT (BWSTT), and robot-assisted TT (RATT) in persons with multiple sclerosis (MS), with focus on gait-related outcome measurements. *Method*. Electronic databases (Pubmed, Pedro, Web of Science, and Cochrane Library) and reference lists of articles and narrative reviews were searched. Pre-, quasi- and true-experimental studies were included if adult persons with MS were involved in TT, BWSTT, or RATT intervention studies published before 2012. Descriptive analysis was performed and two researchers scored the methodological quality of the studies. *Results*. 5 true- and 3 preexperimental studies (mean quality score: 66%) have been included. In total 161 persons with MS were involved (TT, BWSTT, or RATT, 6–42 sessions; 2–5x/week; 3–21 weeks). Significant improvements in walking speed and endurance were reported. Furthermore, improvements of step length, double-support time, and Expanded Disability Status Scale were found. *Conclusions*. There is a limited number of published papers related to TT in persons with MS, concluding that TT, BWSTT, and RATT improve the walking speed and endurance. However, it is not clear what type of TT is most effective. RCTs with larger but more homogeneous populations are needed.

## 1. Introduction

Multiple sclerosis (MS) is a disease causing a widespread degeneration of the central nervous system which gradually results in severe neurological deficits [1]. This neurodegenerative autoimmune disease has a high risk (incidence ranges from 0.1 to 0.2%) in the United States, Canada, Russia, Israel, Europe, New Zealand, and parts of Australia [2]. The patterns of symptoms are complex, variable, and unpredictable [1], translating to extreme debilitation for some, where others conduct their daily lives with no dramatic changes [2]. The variable distribution of demyelization and axonal loss may lead to disorders of strength, sensation, coordination and balance, as well as visual, cognitive, and affective deficits, which may lead to severe progressive limitations of functioning in daily life [3]. The motor problems include muscle weakness, partial or full paralysis, stiffness, slurred speech, twitching muscles, tremor, and spasticity [2]. Locomotor disability in persons with MS can be considered as an emergent characteristic deriving from several mechanisms of functional impairments, including coordination of posture and gait [4, 5]. Physical impairments strongly influence the level of independence that a person with MS is able to achieve [6]. Compton and Coles (2008) described the different signs and symptoms in function of the different affected sites of the nervous system (cerebrum, optic nerve, cerebellum and cerebellar pathways, brainstem, spinal cord, and other sites) [7]. These various symptoms can appear in each of the four clinically defined phenotypes of MS: relapsing-remitting (RR) MS (approximately 55% of the cases), secondary progressive (SP) MS (approximately 30% of the cases), primary progressive (PP) MS (approximately 10% of the cases), progressive relapsing (PR) MS (approximately 5% of the cases). Each type has its own features and progression [2, 8].

Because of the wide variety of symptoms the rehabilitation process in persons with MS is complex and should be multidisciplinary and personspecific. Approximately 75% of the persons with MS experiences mobility problems [9, 10], such as a reduced walking ability [4]. Persons with MS frequently show changes and a greater variability in lower limb kinematics during gait such as reduced stride length and prolonged double limb support time, and in walking speed compared with healthy controls [11, 12]. There is a correlation between strength reduction, especially of the hamstrings, and gait impairment, whatever the clinical type of MS [4].

One of the primary aims of rehabilitation for persons with MS is to increase their levels of activity and participation and so increase their independence [3]. Many factors (physical, cognitive, emotional, and patient's starting profile of impairment and disability) may determine the rehabilitation benefit [6]. Gait rehabilitation is an important part of the therapy to reach the previous goal. Because gait problems can lead to an increased risk of falling, it is important to include balance and walking training in the therapy program [13]. Physiotherapy in chronic MS patients is associated with improved mobility compared with no treatment [10, 14, 15], but the benefit may only last a few weeks [14, 15].

Beside over-ground gait training, different types of gait rehabilitation on a treadmill are possible, such as, treadmill training (TT) with manual assistance and support, treadmill training in combination with body weight support (BWS), and treadmill training with BWS in combination with robot assistance (RA). Since the late 1990s robot-assisted gait rehabilitation has become popular in neurological rehabilitation. Different systems are commercially available, including the “Lokomat” [16, 17] and the “Gait trainer” [18]. Actually TT, with or without BWS and/or RA, is a frequently used technique for gait rehabilitation in neurological diseases such as spinal cord injury (SCI), stroke, and in Parkinson's disease [19–26]. In these populations of patients the results are fairly good, for example the patients walk more symmetrically with higher velocities resulting in a facilitation of the paretic muscles and have a more efficient gait [19]. But, it is unclear which type of gait rehabilitation therapy is the most effective [21, 22, 27].

At the moment, no systematic reviews were found about gait-related outcome measurements in TT in persons with MS. This systematic literature review focuses on the effectiveness of TT with or without BWS and/or RA in persons with MS, measured with gait-related outcome measurements. The research questions of this paper are (1) do persons with MS improve in gait-related outcome measurements (walking speed and endurance, Expanded Disability Status Scale (EDSS-score) and gait parameters) after TT, with or without BWS and/or RA?; (2) is any of the therapies (TT, BWSTT or RATT) superior to the other therapies in terms of gait-related outcome measurements?; (3) what are the long term-effects?

## 2. Methods

A computerized search was conducted for English, French, and Dutch articles published before 2012. The electronic databases PubMed, Web of Sciences, Cochrane Library, and Pedro were investigated. Keywords and MeSH-terms, and their combinations were organized following the Population, Intervention, Comparison, and Outcome (PICO) model [28] and are reported in Table 1. Also the reference lists of the articles and narrative reviews were scanned for relevant publications.

Included were studies on adult (+19 years) persons with MS. Subjects with MS diagnosis were included, whatever the type, grade, or duration of their disease. Effect studies on TT, with or without BWS and/or RA with the primary aim of improving gait function and which encompassed gait-related outcome measurements, were included. Excluded were studies where TT was used in combination with other interventions than BWS and RA. Studies using functional electrostimulation, studies with outcome exclusively focused on physical capacity, electromyographic or kinematic data and/or cardiorespiratory functioning were excluded. Also excluded were animal studies and studies on children. Pre-, quasi- and true-experimental studies were included, with exception of studies only presented as an abstract of a congress.

## 3. Results

**Figure 1:**
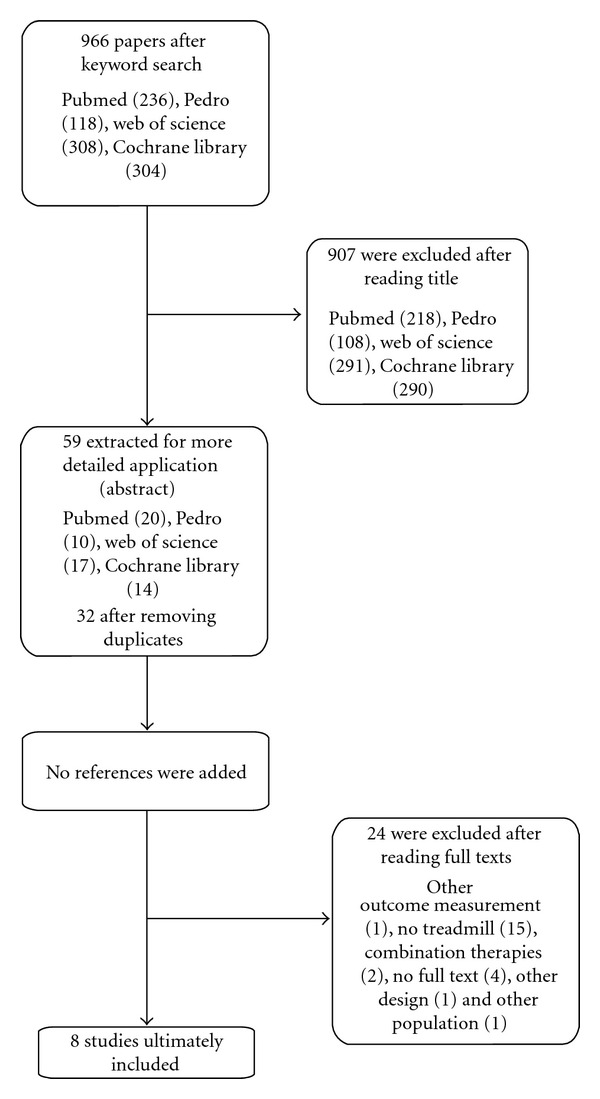
Flowchart of the search strategy.

The flowchart (Figure 1) presents an overview of the search strategy. Ultimately, eight studies were included in this systematic review [29–36]. Five studies were true experimental trials (randomized controlled trials, RCTs [29–33]), no studies were quasi-experimental trials (clinical trials without random assignment) and three studies were preexperimental trials (one study was a randomized trial without comparison group and two studies were case reports with four and six subjects [34–36]) [37]. The methodology checklist: “Evaluation of quality of an intervention study” was used to assess the quality of the included studies [38]. Two researchers scored the studies independently and Cohen's kappa was used to test the interrater reliability. This check-list scores the internal validity of the studies, and consists of seven subscales: study question, study design, subjects, intervention, outcomes, analysis and recommendations. The Cohen's Kappa between the scores of the two researchers was 0.77 (SD 0.13), indicating a good agreement between the scores of the two researchers. The consensus method was used in case of disagreement. In Table 2 an overview of the scores of the Methodology checklist is presented. All studies scored between 48 and 79% on the methodological checklist, with the highest scores for the RCTs (between 62.5 to 79%) and the lowest scores for the case reports (48 and 54%). The mean score was 31.5/48 (SD 5.8) or 66%. We decided to includ all the eight studies. The lower scores were mainly caused by poor quality of the study design, the intervention, and the analysis of the results.

A total of 161 persons with MS participated in the different studies (patients characteristics, e.g., gender, EDSS score, age, and type MS were reported in Table 3). Two studies measured the same subjects, although they described different measurement outcomes and measured the outcome on different moments during and after training [33, 34]. Hence, 145 different persons with MS participated in the studies. In total 34 drop-outs were described. Beer et al. (2008) described five drop-outs in the RATT group which two were directly related to treatment (skin irritation by the fixation belt at the knee/lower leg with full recovery), and there was one drop-out in the conventional walking therapy (CWT) group not related to the treatment [29]. In the study of Vaney et al. (2011) eighteen drop-outs were described, eight in the RATT group (family reason, too weak, did not want to continue, problem with catheterization) and ten in the walking group (language problems, fracture before rehabilitation, too tired, did not want to continue, MS exacerbation) [30]. Of the nineteen subjects in the studies of Newman et al. (2007) and van den Berg et al. (2006) three dropped out for reasons unrelated to training or their MS [33, 34]. Schwartz et al. (2011) described four drop-outs after four weeks treatment, one in the CWT group (patients: uncooperative with treatment) and three in the RATT group (two patients: uncooperative with treatment, one patient participated in another study). They reported four more drop-outs after three months followup and six more drop-outs after six months followup [31]. More females (63% without drop-outs) compared to males participated in the studies. One study did not reported the gender of their participants [30]. If reported no subject had a relapse within eight weeks [33, 34], three months [29–31], six months [32], or one year [36].

Different protocols of TT were reported among the included studies (Design, type, walking speed, level of BWS, and duration and frequency of the interventions were reported in Table 3).

All included studies measured walking speed: two studies using the timed 25-foot walk (T25 FW) [32, 35], one study the twenty-meter walk test (20 MWT) [29], and four studies the ten-meter walk test (10 MWT) [31, 33, 34, 36]. One study uses the 10 MWT and the three-minutes walk test (3 MWT) to measure walking speed [30]. Walking endurance was measured in all the selected studies, except two [30, 35]. In three studies the walking endurance was measured with the six-minute walk test (6 MWT) [29, 31, 32, 36] and in two studies with the two-minute walk test (2 MWT) [33, 34]. The tests were recorded during over-ground walking with exception of one study in which the 6 MWT was done on a treadmill [32]. Two RCTs [31, 32] and the two case reports [35, 36] used the EDSS score to measure the disability. Beer et al. (2008) measured the stride length and Newman et al. (2007) measured the stride length, the cadence, gait cycle time, and foot contact time by using the GAIT-Rite mat [39]. We calculated the effect size (Cohen's d) if the means and standard deviations of the results in the different included studies were reported (Table 4).

### 3.1. Research Question 1: Do Persons with MS Improve in Gait-Related Outcome Measurements (Walking Speed and Endurance, EDSS-Score, and Gait Parameters) after TT, with or without BWS and/or RA?

#### 3.1.1. Walking Speed

In the eight studies that reported walking speed an improvement was measured after training in almost all subjects. Pilutti et al. (2011) reported that during training the walking speed on the treadmill increased significantly (*P* < 0.001) from 1.1 ± 0.10 kmph (0.31 m/s) to 1.6 ± 0.09 kmph (0.44 m/s), and a mean change of 18% on the T25-FW was measured [35]. In the controlled trial of Giesser et al. (2007) one subject who could not walk the 10 MWT before completed the test after training. All subjects who could perform the task could walk faster over ground after training as compared with before training [36]. Lo and Triche (2008) reported a decrease in average time for the T25FW after training. Total change by the end of twelve sessions showed a 31% improvement in the T25 FW. Effect size calculations show that there was a small effect in the Lokomat-BWSTT group at the different measurement times and a large effect in the BWSTT-lokomat group but only when comparing the data on baseline with the data after the BWSTT training sessions [32]. Beer et al. (2008) reported that the effect sizes of differences between RATT and CWT showed a large effect (>0.6) for walking velocity on the 20 MWT. A pre-post-within group analysis revealed a significant improvement of walking speed in both groups (RATT: 0.21 m/s (range 0.09–0.27) to 0.27 m/s (range 0.15 to 0.49), *P* = 0.003 and CWT: 0.24 m/s (range 0.17 to 0.28) to 0.31 m/s (range 0.19 and 0.42), *P* = 0.026). They reported that after a follow-up period of six months the outcome values had returned to baseline in both groups [29]. Van Den Berg et al. (2006) described that after seven weeks individuals of the trained group significantly improved their 10 MWT more, compared with the untrained group (*P* < 0.05). At week twelve, after a four-week rest period, walking performance returned towards baseline scores [33]. Newman et al. (2007) reported that the mean 10 MWT time reduced from 15.6 seconds (SD: 5.6, range 7.8–28.1) to 13.9 seconds (SD: 5.3, range 7.5–27.0), *P* = 0.016 [34] after TT. In the study of Schwartz et al. (2011) at the end of the treatment only in the CWT group, and not in the RATT group, a significant improvement (small effect size) on the 10 MWT was found (mean change from baseline: 0.1 m/s, SD 0.2) [31]. Vaney et al. (2011) show improvements (small effect size) on the 10 MWT and the 3 MWT in the walking group (10 MWT: 0.09 m/s, SD 0.17, 95% CI: 0.01 to 0.16, 3 MWT: 0.11 m/s, SD 0.17, 95% CI: 0.04 to 0.18) and in the RATT group (10 MWT: 0.03 m/s, SD 0.09, 95% CI: −0.00 to 0.07, 3 MWT: 0.03 m/s, SD 0.10, 95% CI: −0.01 to 0.07) [30].

#### 3.1.2. Walking Endurance

 In all six studies that reported walking endurance an improvement was measured after training in almost all subjects. One case report showed that three out of four subjects showed improvement in endurance as measured with the 6 MWT and two subjects who could not walk the 6 MWT before completed the test after training [36]. Of the thirteen subjects of Lo and Triche (2008) all but one person had an improvement in distance covered in the 6 MWT after training. The total change by the end of the twelve sessions showed a 38.5% improvement for the 6 MWT on the treadmill (large effect in the Lokomat-BWSTT group and a moderate effect in the BWSTT-Lokomat group) [32]. Comparing RATT and CWT showed a moderate effect for the 6 MWT distance, favoring RATT (RATT: 74 m (range 34–97) to 81 m (range 44–137), *P* = 0.006/CWT: 87 m (range 62–101) to 83 m (range 64–145), *P* = 0211) [29]. The trained and untrained subjects in the study of van den Berg et al. (2006) significantly improved their 2 MWT times after seven weeks and after week twelve the walking performance returned towards baselines scores. A small effect was calculated in the delayed training group [33]. The walking endurance of the subjects in the study of Newman et al. (2007) increased from a mean 88.2 m (SD: 32.2, range 44.6–154.0 m) to 94.3 m (SD: 32.2, range: 55.2 to 156.1 m), *P* = 0.020 on the 2 MWT distance [34]. Schwartz et al. (2011) reported at the end of the treatment a significant improvement (small effect size) on the 10 MWT in the CWT group (mean change from baseline 30.2 m, SD: 37.6) but not in the RATT group.

#### 3.1.3. EDSS Score

Four studies measured an improvement in EDSS score after training: Lo and Triche (2008) in eleven of the thirteen subjects (baseline scores between 7 and 3.5 and after training a gain from 0.5 to 2 points, *P* = 0.001, large effect size) and Pilutti et al. (2011) and Giesser et al. (2007) both in only one subject (both a decrease of 0.5 after training). In this last case study it was reported that the subject who did improve in EDSS score (baseline: 7.0-after training: 6.5) started from a higher functional level and was less neurologically impaired in terms of muscle strength and balance than the other three subjects [36]. Schwartz et al. (2011) described that at the end of the treatment both RATT and CWT groups showed a significant improvement in EDSS score (moderate effect). In the RATT the mean change from baseline was −0.29 (SD 0.4, pre: 6.2 SD 0.5; post: 5.9 SD 0.6) and in the CWT −0.31 (SD 0.3, pre: 6.0 SD 0.6; post: 5.7 SD 0.7) [31].

#### 3.1.4. Gait Parameters

A decrease in double support time in twelve of the thirteen subjects (*P* = 0.03), and a trend toward greater normalization of the double support time for those randomized to the treadmill group compared to the robot group (−5.9% versus −1.9%, *P* = 0.06) (moderate effect size) was found in the study of Lo and Triche (2008). Furthermore these authors found no significant changes in step length ratio after treatment nor significant differences between the two groups for step length ratio (0.004 versus 0.02) [32]. Newman et al. (2007) reported no significant difference in cadence after training. A significant increase in swing phase time of the weak leg (baseline: 33% ± 9.3 (range: 1.7 to 42.7), posttraining: 36% ± 4.5 (range: 26.7 to 41.7); *P* = 0.03, small effect size), a significant decrease in stand phase time of the weak leg (baseline: 67% ± 9.3 (range: 57.3 to 98.3), posttraining: 63.8% ± 4.5 (range: 58.3 to 73.4) *P* = 0.03, small effect size) and a significant increase in stride length of the strong leg (Baseline: 98.7% ± 21 range: 65.5 to 135.0, posttraining: 104.0% ± 21 range: 75.4 to 146.0; *P* = 0.04) were described [34]. Beer et al. (2008) reported a nonsignificant small effect (effect size 0.2 to 0.4) for stride length [29].

### 3.2. Research Question 2: Is Any of the Therapies (TT, BWSTT, Or RATT) Superior to the Other Therapies in Terms of Gait-Related Outcome Measurements?

Four studies (four RCTs) compared different types of gait training. Three studies compared RATT with over-ground CWT and one study RATT with BWSTT. Comparing CWT with the RATT, Beer et al. (2008) found in their RCT a higher benefit in walking velocity and knee extensor strength by RATT compared to CWT. They concluded that RATT treatment might actually be an effective treatment option for the subgroup of persons with MS with severe walking disabilities [29]. The study of Schwartz et al. (2011) reported that they found no significant differences in mean and mean change from baseline of gait parameters and EDSS score between the RATT and CWT in both groups [31]. The RCT of Vaney et al. (2011) found a weak evidence that the walking group improved more in the 3 MWT and the 10 MWT compared with the RATT group. But, after correction for multiple testing there were no significant between-group differences [30]. The RCT of Lo and Triche (2008) based on a cross-over design for BWSTT and RATT reported significant within-subjects improvements but no significant differences between the treatment groups [32].

### 3.3. Research Question 3: What Are the Long Term-Effects?

Van den Berg et al. (2006) described that after twelve weeks, including a four-week rest period, the walking performance returned toward baseline scores [33]. One RCT measured followup data after six months and reported that the outcome values returned to baseline in both groups (RATT and CWT) [29]. The RCT of Schwartz et al. (2011) did a followup at three and six months. At three months, the 6 MWT and EDSS improved significantly only in the CWT group and the 10 MWT had not changed in either group. At six months the 6 MWT, 10 MWT, and EDSS scores are returned to baseline scores in both groups. After calculating the effect score a small effect was found for EDSS after six months [31].

## 4. Discussion

With this paper it can be established that only a few studies have been published that investigated gait-related outcome measurements, such as walking speed and endurance, step length and EDSS score, after TT with or without BWS and/or RA, in persons with MS. The results suggest that TT, BWSTT, and RATT improve the walking speed and maximal walking distance in persons with MS, but it is not clear what type of TT intervention is the most effective.

This systematic review included pre-, quasi-, and true-experimental study designs. The methodological quality of the included studies was good, with five RCTs with a quality score between 62.5% and 79%, although three of the eight included studies are preexperimental studies and therefore of lower methodological quality. The number of subjects is rather limited. This low number of participants is probably due to the practical feasibility of the intervention and the difficulty in selecting and motivating the patients. Different types of MS (RR, PP, SP, PR) and subjects with different gradations of gait problems were selected in the included studies. The information on the EDSS score, type of MS, and/or severity of gait problems was limited in some studies or even not reported. Also the severity of the symptoms at baseline was different in the subjects of the different studies. The selection of the patients for intervention studies is difficult in the MS population because of the variability in symptoms, the different types of MS, and the different and unpredictable course of the disease [1].

The results, in terms of gait-related outcome measurements, were promising with improvements in walking speed and endurance. Hence, when comparing RATT with BWSTT no significant differences between these two methods were reported. In the study of Lo and Triche (2008) some higher effect sizes were measured after BWSTT compared to RATT. Vaney et al. (2011) reported that the between-group difference was in favor of the CWT and not of the RATT. However, it is not clear what type of TT intervention is most effective with respect to gait-related outcome measurements. Furthermore, the long-term benefits of TT are not sufficiently studied at the moment. Only two studies included an acceptable follow-up period of six months and reported that the outcome values returned to baseline.

The positive outcome on gait speed and endurance is important for the clinical practice. Also clinically interesting is the finding of Giesser et al. (2007), who included only severely affected persons with MS (EDSS 7–7.5). After about twenty BWSTT sessions, respectively, one and two persons who could not complete the tests before the training completed the 10 MWT and the 6 MWT after training [36]. This means a large impact on these patients' autonomy. Also interesting is the gain of 0.5 or one point in EDSS score measured in a few patients [35, 36].

In other neurological populations, such as Stroke, Parkinson's disease, and SCI, more studies were conducted on this topic and a few systematic reviews summarize the results [19, 21–23, 26, 27, 40]. These results are similar to the results of this paper. Some positive effects are reported on gait parameters, but it is unclear which therapy modality is most effective. In acute and subacute patients after stroke (more than in patients treated more than three months after stroke), the use of RATT interventions in combination with physiotherapy increased the chance of regaining independent walking ability for patients, but it was not associated with improvements in walking velocity nor walking capacity [40]. In Parkinson's disease TT improved gait speed, stride length, walking distance, but cadence did not improve at the end of study [26]. Also in SCI populations there are some positive results on gait-related outcome measurements, but there is insufficient evidence to conclude that any approach to locomotor training is more effective than any other for improving the walking function of people with SCI [22, 27]. Comparable to the results in persons with MS in any of these other neurological disorders, the long-term effects of the different therapies are not clear at the moment.

We included studies that used TT in its different forms (TT, BWSTT, and RATT). We are aware that different types of walking training lead to differences in biomechanics and physiology [41–44]. Riley et al. (2007) have shown that treadmill gait is qualitatively and quantitatively similar to over-ground gait. Only small differences in kinematic and kinetic parameters can be detected with magnitudes within the range of repeatability of measured kinematic parameters [44]. Hence, in healthy elderly subjects there was a significantly increased cadence and decreased stride length and stride time, along with reduced joint angles, moments, and powers measured during treadmill walking compared to over-ground walking [43]. More differences in kinematics occurred when comparing TT with BWS and RA. Differences among BWSTT and full weight bearing TT are a raised center of gravity, leading to limited downward excursion, a decreased percentage of stance, total double-limb support time, hip and knee angular displacement, and an increased single-limb support time. Other adaptations to BWS were a reduced mean burst amplitude in muscles required for weight acceptance (i.e., erector spinae and gluteus medius muscles) and push-off (i.e., medial gastrocnemius muscle) and an increase in mean burst amplitude in the investigated muscle that is active during swing (i.e., tibialis anterior muscle) [45]. Higher levels of BWS increased lower extremity kinematic variability, with more variability at the hip joint for older subjects [41]. Although Hidler et al. (2008) described that the overall kinematics in the Lokomat are similar to those on a treadmill, there was significantly more hip and ankle extension and greater hip and ankle range of motion during walking in the Lokomat (*P* < 0.05) measured [42].

The initial percentage BWS used in the included studies was very high in some cases, if reported the percentage of unweighting was from 30% to more than 80% of the body weight. Because the gait patterns (temporospatial and kinematic changes) are significantly changed by 50% and 70% BWS [46, 47] and also changes in EMG activity of the muscles occur when walking at these high percentage of BWS [48] we can doubt the effect on functional gait of training in such high percentages of BWS.

Furthermore, the effort the patients make, not to mention the effort of the therapists and the number of therapist needed to assist the patients, varies in the different types of TT. The preparation of the patients for RATT is a time intensive process, but once they are installed in the robot only one therapist is needed to assist the patient. TT or BWSTT in subjects with severe impairments implies a highly personalized and labor-intensive task for the therapist such as in the study of Giesser et al. (2007) [36]. The number of therapists needed to accomplish the treatment during TT was significantly higher than during RATT intervention with the LokoHelp in the study of Freivogel et al. (2009) [49]. This meant a 25% increase in staff requirements [49]. Because of the limited number of studies in persons with MS on this topic we have chosen to include the different types of TT but selected on specific gait-related outcome measurements.

Other issues are the physiological and psychological effects of training. Several studies reported the positive effects of TT on functional mobility, cardiovascular fitness, quality of life, and very significant health-related benefits that may decrease the risk of secondary health complications associated with physical inactivity [50–53]. Also wheel chair-dependent individuals enjoy and value the normalizing experience of seeing themselves upright and participating in the walking motion [50].

It is necessary that large RCTs compare the different types of TT with no training in persons with MS with subdivisions depending on the type of MS and the degree of independence to get a clear idea about the effectiveness of the different types of TT.

## 5. Conclusions

We can conclude that actually there is a limited amount of literature related to TT, BWSTT, and RATT in persons with MS, suggesting that TT, BWSTT, and RATT improve the walking speed and maximal walking distance in persons with MS. Although there are some promising results for RATT, it is not clear what type of TT intervention is the most effective. Therefore, RCTs with larger but more homogeneous populations are needed.

## Figures and Tables

**Table 1:** Key words and MeSH terms and their combinations that were used in the literature search. In the search keys: between the columns “AND” was used. The key words “Multiple Sclerosis”, “Gait”, “Walking”, “Exercise”, and “Exercise Therapy” were used as MeSH terms in the database PubMed.

P: population	I: intervention	C: comparison	O: outcome
Multiple sclerosis	(Walking) OR (gait) OR (step) AND (robot-assisted) OR (body weight support) OR (body weight supported) OR (partial body weight) OR (partial body weight supported) OR (weight-support) OR (weight-supported) OR (treadmill) OR (motorized rehabilitation) OR (motorized training) OR (automatic orthoses) OR (locomotor rehabilitation) OR (locomotor training) AND (exercise) OR (exercise therapy) OR (training) OR (rehabilitation)	Conventional therapies	(walking speed) OR (gait speed) OR (walking distance) OR (walking capacity) OR (walking endurance) OR (stride length) OR (step length)

**Table 2:** Overview of the scores (after agreement between the two researchers, Cohen's Kappa: 0.77 (SD 0.13, good agreement)) of the methodological checklist. The mean quality score of the studies is 31.5/48 (SD 5.8).

	Beer et al. (2008) [29] (True-exp)	Vaney et al. (2011) [30] (True-exp)	Schwartz et al. (2011) [31] (True-exp)	Lo and Triche (2008) [32](True- exp)	Van den Berg et al. (2006) [33] (True-exp)	Newman et al. (2007) [34] (Pre-exp)	Pilutti et al. (2011) [35] (Pre-exp)	Giesser et al. (2007) [36] (Pre-exp)	Mean scores
Study question (/2)	2	2	2	2	1	2	2	2	1.88
Study design (/14)	12	10	12	10	12	8	6	6	9.50
Subjects (/8)	5	6	4	6	4	4	4	5	4.75
Intervention (/6)	3	4	4	3	3	2	2	1	2.75
Outcomes (/6)	6	5	6	5	5	3	3	5	4.75
Analysis (/10)	8	8	7	6	4	6	7	2	6.00
Recommendations (/2)	2	2	2	2	1	2	2	2	1.88
Total quality score (/48)	38 (79%)	37 (77%)	37 (77%)	34 (71%)	30 (62.5%)	27 (56%)	26 (54%)	23 (48%)	31.5 (SD5.8) (66%)
Cohen's Kappa between two raters	0.83 (VG)	0.74 (G)	0.83 (VG)	0.85 (VG)	0.51 (M)	0.68 (G)	0.92 (VG)	0.78 (G)	0.77 (SD0.13) (G)

exp: experimental, SD: standard deviation, VG: very good agreement, G: good agreement, M: moderate agreement.

**Table 3:** Descriptive analysis of the included studies structured following the PICO method.

Reference.		Participants					Intervention (+Comparison)				Outcome		Results
Author (year)	Study (MQS)	Number	Male/female	EDSS score	Age	Type MS	Design	Type	Speed/BWS	Sessions	Outcome-measurements	Assessment times	Gait-related outcome measurements
Beer et al. (2008) [29]	true exp. (79%)	29 (35: 6 drop-outs; 5 in RATT and 1 in CWT)	12 m/23 f RATT: 7 m/12 f, CWT: 5 m/11 f	RATT: 6.5 (range: 6–7.5), CWT: 6.5 (range: 6–7.5)	RATT: 49.7 (SD 11), CWT: 51 (SD 15.5)	Chronic P, RR	Prospective RCT, comparing RATT with CWT	RATT: BWSTT + Lokomat (*n* = 19 → *n* = 14), CWT: conventional walking training (*n* = 16 → *n* = 15)	RATT: initial BWS (40–80%), assistance of leg movements (40–100%) and speed (1–1.5 kmph/0.28–0.42 m/s). ↓ BWS and assistance and ↑ speed. CWT: walking over ground with or without walking aids, with assistance.	15 sessions, 30 min, 5x/w	Walking speed (20 MWT), walking endurance (6 MWT), stride length (cm)	Baseline, after 3w and at followup after 6 m.	After RATT: sign. ↑ walking speed and endurance. After CWT: sign. ↑ walking speeds. No sign. differences between groups. Followup (*n* = 23): outcome values returned to baseline.

Vaney et al. (2011) [30]	true exp. (77%)	49 (67: 18 drop-outs)	?	RATT: 5.9 (SD 0.90, range: 3–6.5) CWT: 5.7 (SD 1.06, range: 3–6.5)	RATT: 58.2 (SD 9.42, range: 37–73) CWT: 54.2 (SD 11.28, range: 36–74),	?	RCT comparing RATT with CWT	RATT: BWSTT + Lokomat (*n* = 26), CWT: walking in group with physiotherapist (*n* = 23)	RATT: initial BWS 50%, individually adapted, speed: regulated on gait observation, initial guidance 100% and reduced as much as possible. CWT: in gym room or outside on uneven ground + walking aids.	RATT: 6 to 10 (mean 9) sessions, CWT: 7 to 10 (mean 8) sessions. 30 min.	Walking speed (10 MWT, 3 MWT on 80 m hallway)	Baseline, after treatment.	In both groups: ↑ walking speed. No sign. between-group differences. sign. ↑ walking speed, between group difference was in favor of the CWT.

Schwartz et al. (2011) [31]	true exp. (77%)	28 (32: 4 drop outs; 1 in CWT, 3 in RATT) Follow-up 3 m *n* = 24, 6 m *n* = 18	14 m/18 f RATT: 7 m/8 f, CWT: 7 m/10 f	RATT: 6.2 (SD 0.5, range: 5.5–7), CWT: 6 (SD 0.6, range: 5–7)	RATT: 46.8 (SD 11.5, range: 29–69), CWT: 50.5 (SD 11.5, range: 28–70)	RP, SP, PP	Prospective RCT	RATT: BWSTT + Lokomat (*n* = 15), CWT: gait and dynamic balance exercises, standing from sitting training and walking with or without walking aids (*n* = 17).	RATT: initial BWS: 40%, after 2 w 30%, after 4 w 20%. speed: maximum speed tolerated.	12 sessions, 30 min, 2–3x/w for 4 w	Walking speed (10 MWT), walking endurance (6 MWT), disability (EDSS)	Baseline, after 4 w, followup after 3 and 6 m.	After CWT: sign.↑ walking endurance and speed and ↓ EDSS. After RATT: no sign. ↑ walking endurance and speed, sign. ↓ EDSS. Followup: outcome values returned to baseline.

Lo and Triche (2008) [32]	true exp. (71%)	13	7 m/6 f	4.9 (SD 1.2)	49.8 (SD 11.1)	RR SP (*n* = 8), PP (*n* = 5)	prospective randomized pilot study, randomized cross-over design, RATT-BWSTT (*n* = 6), BWSTT-RATT (*n* = 7)	(1) BWSTT followed by BWSTT + Lokomat or (2) BWSTT + Lokomat followed by BWSTT. After 1st phase (T2), 6-week washout period (T3), crossed-over to the alternate treatment.	Initial BWS: 30% to 40%, initial speed: 1.5 kmph (0.42 m/s). ↑ speed to 2.2 to 2.5 kmph (0.61 to 0.69 m/s) before ↓ BWS	12 sessions (6/phase), 40 min, 2x/w	Walking speed (T25 FW), walking endurance (6 MWT on treadmill), disability (EDSS)	T1 (baseline), T2 (after first phase, 3 w), T4 (end study, 12 w)	↑ walking speed, ↑ walking endurance (*n* = 12), ↓ double limb support time (*n* = 12). ↓ EDSS. No sign. differences due to treatment order.

van den Berg et al. (2006) [33]	true exp. (62.5%)	16 (19: 3 drop outs)	3 m/13 f	? (able to walk 10 m (using aids if required) in less than 60 s + could walk safely on treadmill without therapist or BW support.	IT: 30–65, DT: 30–65	?	RCT, pilot study (random.) cross over design.	TT, IT: training-no training (*n* = 8); DT: no training-training (*n* = 8).	Walking duration ↑ as tolerated, up to max 30 min with a max of 3 rest periods. Once max walking duration was attained, intensity ↑ by ↑ speed. Encouraged: 55–85% of age-predicted max HR.	12 sessions, 30 min, 3x/w	Walking speed (10 MWT), walking endurance (2 MWT)	Baseline, week 7 (T1) and 12 (T2)	At T1: ↑ walking endurance; trained group sign. ↑ walking speed compared to untrained group. At T2: walking performance returned toward baseline scores.
Newman et al. (2007) [34]	pre-exp. (56%)				53.6 (SD 8.67, range: 30–65)		RT, repeated measures trial with blinded assessments	TT	At 55–85% of age-predicted max HR. Speed ↑ as directed by participants once able to walk for 30 min continuously.		Cadence, gait cycle time, foot contact time, and stride length (Gait-Rite mat), walking speed (10 MWT), and walking endurance (2 MWT)	Baseline and after 4 w	Sign. ↑ walking speed and endurance. Weak leg sign. ↑ swingphase time and ↓ standphase time. Strong leg sign. ↑ stride length.

Pilutti et al. (2011) [35]	pre-exp. (54%)	6	2 m/4 f	6.9 (SD 1.07) range: 5.5–8.0	48.2 (SD: 9.3)	PP (*n* = 5), SP (*n* = 1)	Before-after trial	BWSTT	Baseline: 1.1 ± 0.10 kmph (0.31 m/s) with 77.9% ± 10.76% BWS. During training ↑speed and endurance and ↓BWS	36 sessions, 30 min, 3x/w	Walking speed (T25 FW), disability (EDSS)	Baseline, after 12 w	↓0.5 EDSS (*n* = 1), ↑ walking speed.

Giesser et al. (2007) [36]	pre-exp. (48%)	4	1 m/3 f	7.0–7.5	42, 44, 48, 54	SP	Case series, intervention study	BWSTT + 3 trainers each subjects (1 left leg, 1 right leg, 1 trunk/pelvis)	Initially: BWS at maximum level (knee buckling and trunk collapse can be avoided during stepping). BWS ↓ if able to support their weight during stepping at normal speeds (0.85–1.03 m/s)	39, 40, 42, 42 sessions, 2x/w	Disability (EDSS), walking speed (10 MWT), walking endurance (6 MWT)	Baseline and after training	↓ EDSS (*n* = 1), ↑ walking speed (*n* = 4), could not walk before and complete 10 m after (*n* = 1), ↑ walking endurance (*n* = 3), could not walk before and complete 6 min after (*n* = 2).

exp.: experimental design, MQS: methodological quality score, RATT: robot-assisted gait training, CWT: conventional walking therapy, SD: standard deviation, IT: immediate training, DT: delayed training, PP: primary progressive, SP: secondary progressive, RR: relapsing remitting, P: progressive, RT: randomized trial, BWS: body weight support, RCT: randomized controlled trial, BWS: body-weight support, HR: heart rate, sign.: significant, EDSS: expanded disability status scale, T-25 FW: timed 25-foot walk, 6 MWT: six-minute walk test, 2 MWT: two-minute walk test, three MWT: three-minute walk test, 20 MWT: twenty-meter walk test, 10 MWT: ten-meter walk test, Sign.: significant, ↑: increase, ↓: decrease.

**Table 4:** Effect size calculations (Cohen's d).

	Outcome	Baseline mean ± SD	After training mean ± SD			Effect size (Cohen's d)
	RATT group					
	10 MWT	0.52 ± 0.32	0.57 ± 0.34			0.151
Vaney et al. (2011) [30]	3 MWT	0.58 ± 0.38	0.61 ± 0.41			0.076
	CWT group					
	10 MWT	0.6 ± 0.34	0.69 ± 0.41			0.239*
	3 MWT	0.65 ± 0.37	0.76 ± 0.43			0.274*
			T2 (week 4)	T3 (month 3)	T4 (month 6)	
	RATT group					
	EDSS	6.2 ± 0.5	5.9 ± 0.6	6.0 ± 0.7	6.0 ± 0.76	T1-T2: 0.543** T1-T3: 0.329* T1-T4: 0.311*
	10 MWT	0.49 ± 0.3	0.45 ± 0.3	0.46 ± 0.3	0.47 ± 0.3	T1-T2: 0.133 T1-T3: 0.100 T1-T4: 0.067
	6 MWT	125.8 ± 74.7	133.4 ± 85.1	120.3 ± 84.9	121.1 ± 82.1	T1-T2: 0.095 T1-T3: 0.069 T1-T4: 0.060

Schwartz et al. (2011) [31]	CWT group					
	EDSS	6.0 ± 0.6	5.7 ± 0.7	5.7 ± 0.7	5.8 ± 0.6	T1-T2: 0.460** T1-T3: 0.460** T1-T4: 0.333*
	10 MWT	0.53 ± 0.31	0.63 ± 0.4	0.6 ± 0.4	0.5 ± 0.3	T1-T2: 0.279* T1-T3: 0.196 T1-T4: 0.098
	6 MWT	151.5 ± 92.0	175.7 ± 119.0	160.7 ± 118.0	140.0 ± 116.4	T1-T2: 0.228* T1-T3: 0.087 T1-T4: 0.110
	EDSS	4.9 ± 1.2	3.9 ± 0.7			1.018***
			T2 (week 7)	T4 (week 12)		
	Lokomat-BWSTT					
	T25 FW	8.8 ± 3.1	7.4 ± 3.8	6.6 ± 2.3		T1-T2: 0.404* T2-T4: 0.255* T1-T4: 0.806***
	6 MWT	166 ± 57.6	217.3 ± 65.9	249.2 ± 98		T1-T2: 0.829*** T2-T4: 0.382* T1-T4: 1.035***
	DST	30.1 ± 5.4	28.4 ± 7.4	26 ± 6.4		T1-T2: 0.262* T2-T4: 0.347* T1-T4: 0.692**

Lo and Triche (2008) [32]	BWSTT-Lokomat					
	T25 FW	10.9 ± 5	6.8 ± 3	7 ± 3.6		T1-T2: 0.994*** T2-T4: 0.060 T1-T4: 0.895***
	6 MWT	266.9 ± 102	339 ± 135.8	350.4 ± 124		T1-T2: 0.600** T2-T4: 0.088 T1-T4: 0.735**
	DST	35.8 ± 9.3	28.7 ± 7.5	29.2 ± 9.7		T1-T2: 0.840*** T2-T4: 0.058 T1-T4: 0.695**

	IT Group					
	10 MWS	17.8 ± 5.4	17.2 ± 6.2			0.103
	2 MWS	71.0 ± 22.8	74.5 ± 33.9			0.121
Van den Berg et al. (2006) [33]	DT Group					
	10 MWS	14.0 ± 5.5	13.1 ± 6.5			0.149
	2 MWS	99.5 ± 30.0	106.8 ± 36.7			0.218*
	10 MWT	15.6 ± 5.6	13.9 ± 5.3			0.312*
	2 MWT	88.2 ± 32.2	94.3 ± 32.2			0.189
	% Time in swing (wk)	33 ± 9.3	36 ± 4.5			0.411*
	% Time in stance (wk)	67 ± 9.3	63.8 ± 4.5			0.438*

Newman et al. (2007) [34]	% Time in swing (str)	33.5 ± 5.1	33.3 ± 7.1			0.032
	% Time in stance (str)	66.5 ± 5.1	66.6 ± 7.1			0.016
	Stride length (str)	98.7 ± 21	104.0 ± 21			0.252
	Stride length (wk)	98.6 ± 21.9	103.2 ± 21.5			0.212
	Cadence	92 ± 21	91 ± 17			0.052

Pilutti et al. (2011) [35]	EDSS	6.9 ± 1.07	6.8 ± 1.03			0.095

effect size calculation (Cohen's d). In two studies the calculation of the Cohen's d was not possible. In Beer et al. (2008) [29] no mean scores and SD were reported and in Giesser et al. (2007) [36] only the individual scores were reported. *Small effect, **Moderate effect, ***Large effect.
